# Adverse drug events (ADEs) risk signal mining related to eculizumab based on the FARES database

**DOI:** 10.3389/fphar.2024.1440907

**Published:** 2025-01-09

**Authors:** Xi-Feng Wang, Lu-Ri Bao, Ta-La Hu, Rui-Feng Xu, Wu-Niri Gao, Jing-Yuan Wang, Jian-Rong Zhao, Zhen-Li Fu, Shu-Fang Wang, Yan Meng

**Affiliations:** ^1^ Department of Nephrology, The Affiliated Hospital of Inner Mongolia Medical University, Hohhot, China; ^2^ Department of Pathology, School of Basic Medicine, Inner Mongolia Medical University, Hohhot, China; ^3^ Department of Hemodialysis, The No. 2 Hospital of Hohhot, Hohhot, China; ^4^ The First Department of Specialty Medicine, Inner Mongolia Corps Hospital of The Chinese People’s Armed Police Force, Hohhot, China

**Keywords:** eculizumab, FAERS, adverse drug events, adverse drug reaction monitoring, ADRM

## Abstract

**Introduction:**

Eculizumab is a C5 complement inhibitor approved by the FDA for the targeted treatment of four rare diseases, paroxysmal nocturnal hemoglobinuria (PNH), atypical hemolytic uremic syndrome (aHUS), generalized myasthenia gravis (gMG), and aquaporin-4 immunoglobulin G-positive optic neuromyelitis optica spectrum disorders (AQP4-IgG+NMOSD). The current study was conducted to assess real-world adverse events (AEs) associated with eculizumab through data mining of the FDA Adverse Event Reporting System (FAERS).

**Methods:**

Disproportionality analyses, including Reporting Ratio Ratio (ROR), Proportional Reporting Ratio (PRR), Bayesian Confidence Propagation Neural Network (BCPNN), and Multi-Item Gamma Poisson Shrinker (MGPS) algorithms were used to quantify the signals of eculizumab-associated AEs.

**Results:**

A total of 46,316 eculizumab-related ADEs reports were identified by analyzing 19,418,776 reports in the U.S. Food and Drug Administration Adverse Event Reporting System (FAERS) database. A total of 461 PTs were identified as satisfying by all four algorithms. These PTs reported adverse reactions consistent with the specifications, such as fatigue, nasopharyngitis, meningococcal infection, fever, and anemia. Some PTs, such as aplastic anemia, gene mutation, mastication disorder, kidney fibrosis, BK virus infection, abnormal neutrophil count, C3 glomerulopathy, neuroblastoma, and glomerulonephritis membranoproliferative, were also detected outside the instructions. The median time to onset of eculizumab adverse events was 159 days (interquartile range [IQR] 11∼738 days). In addition, at the PT level, 51 PTs were determined to have an imbalance in the occurrence of ADEs between the sexes.

**Conclusion:**

These findings provide valuable insights into the occurrence of ADEs following the use of eculizumab and could support clinical monitoring and risk identification efforts.

## 1 Introduction

In the complement pathway, activated C5 releases the allergenic toxins C5a and C5b, which interact with C6-C9 and membrane-inserted compartments to form membrane attack complexes (MACs) that lead to lysis, damage or activation of target cells (Ricklin et al., 2016). Eculizumab (Soliris) is a recombinant humanized monoclonal antibody that specifically binds to the C5-terminal complement and inhibits the cleavage of C5 to C5a and C5b via complement activation (Miyamoto, 2014). It is currently approved by the U.S. Food and Drug Administration (FDA) for the treatment of paroxysmal nocturnal hemoglobinuria (PNH), atypical hemolytic uremic syndrome (aHUS), generalized myasthenia gravis (gMG), and aquaporin-4 immunoglobulin G-positive optic neuromyelitis optica spectrum disorders (AQP4-IgG+NMOSD), which play important roles in the allopathic treatment of complement-associated immune disorders. Eculizumab was introduced introduced to China for treating PNH and aHUS in adults and children on 5 September 2018 (Li-li et al., 2022a). Relevant data suggest that eculizumab treatment leads to a decrease in transfusion dependency, a decrease in the incidence of hemolysis and thrombosis, and an improvement in quality of life (Kelly et al., 2011). Concerning eculizumab biosimilars, two variants are currently available: Bkemv (eculizumab-aeeb) and Epysqli (eculizumab-aagh). On 29 May 2024, the U.S. FDA granted approval to Amgen’s Bkemv as the first biosimilar interchangeable with AstraZeneca’s Soliris (eculizumab), for the reduction of hemolysis in patients with paroxysmal nocturnal hemoglobinuria (PNH) and for the inhibition of complement-mediated thrombotic microangiopathy in patients with atypical hemolytic uremic syndrome (aHUS). Produced by Samsung Bioepis Co. Ltd., Epysqli was initially approved in the European Union, Iceland, Liechtenstein, and Norway on 26 May 2023, and received FDA approval on 19 July 2024. Given that biosimilars may have different therapeutic efficacy and safety profiles, it is important to investigate information about their availability and potential impact.

Despite the promising therapeutic effects of eculizumab, its use since its introduction has been found to increase the risk of certain pathogenic infections, particularly meningococcal infections, for which the FDA black box warns (Crew et al., 2019). Other common adverse reactions include headache, nasopharyngitis, nausea, vomiting, diarrhea, hypertension and upper respiratory tract infection (Nishimura et al., 2023). It is therefore crucial to determine the real-world risk of ADE associated with eculizumab to ensure its safe and rational use.

The Adverse Drug Event (ADE) Spontaneous Reporting System database is a major source for mining signals of adverse drug reactions (REN et al., 2011). The FAERS database, with data from national health workers or patients, reflects to some extent the occurrence of drug ADEs in the real world and can therefore help to uncover adverse reactions that are difficult to detect in premarket clinical studies of drugs (Xu, 2015). Given that the adverse reactions in the eculizumab specification are primarily from clinical trials,we utilized the FAERS database for disproportionality analyses to monitor and evaluate the long-term safety of eculizumab, providing a comprehensive and valuable reference for its safety in the real world.

## 2 Materials and methods

### 2.1 Data sources

We implemented a retrospective pharmacovigilance study using data from the FAERS database from January 2007 to the third quarter of 2023. FAERS can be accessed at https://fis.fda.gov/extensions/FPD-QDE-FAERS/FPD-QDE-FAERS.html. Documents in FAERS describe demographic and management information (DEMO), drug information (DRUG), reporting source (RPSR), preferred terms (PT) for adverse event coding (REAC), patient outcomes (OUTC), therapeutic period of the reported medication (THER), indications for use of medication (INDI), and deleted cases (DELE) (Chen et al., 2022). In this study, all the ASCII packet data for 67 quarters from the first quarter of 2007 to the third quarter of 2023 were extracted and imported into SAS 9.4 software for data cleaning and analysis.

### 2.2 Data processing

**FIGURE 1:**
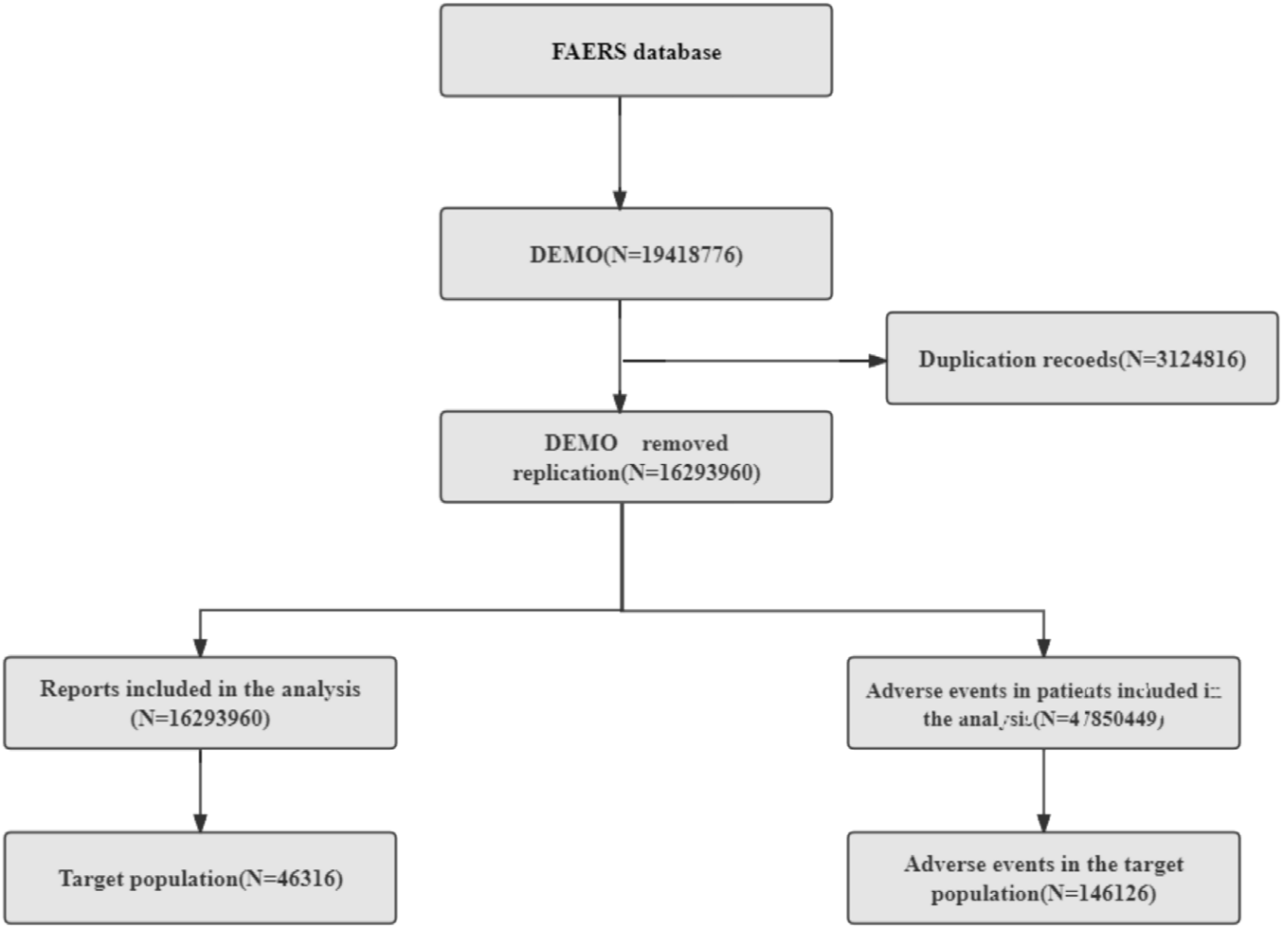
Process for screening eculizumab-related ADEs from the FAERS database.

We screened 19,418,776 patients from the FAERS database. First, we removed duplicate records (3,124,816); selected the PRIMARYID, CASEID, and FDA_DT fields of the DEMO table according to the FDA-recommended method for removing duplicate reports; sorted them by CASEID, FDA_DT, and PRIMARYID; and retained the largest FDA_DT value for reports with the same CASEID, followed by retaining the largest PRIMARYID value for reports with both the same CASEID and FDA_DT. We ultimately included 46,316 reports with eculizumab as the primary treatment and 146,126 cases of adverse events for further analysis (Figure 1). The 3D structure of eculizumab was derived from PubChem (https://pubchem.ncbi.nlm.nih.gov) (Kim et al., 2023).

### 2.3 Data mapping

The most current MedDRA dictionary (MedDRA 26.1) was applied to describe ADEs in the FAERS database in terms of system organ class (SOC) and preferred terms (PT).

Table 1 shows the screening conditions for the target drug population. The field (DRUGNAME) in the FAERS database indicates the name of the drug, and the field (PROD_AI) indicates the product composition. At the same time, the degree of suspicion was limited to the report of the “Primary Suspect Drug (PS)”.

**TABLE 1:** Keywords for screening the target drug population.

Drug name	Drug screening conditions
ECULIZUMAB	INDEX (PROD_AI,“ECULIZUMAB”) OR INDEX (DRUGNAME,“ECULIZUMAB”) OR INDEX (DRUGNAME,“SOLIRIS”)

### 2.4 Data mining

Many organizations have used disproportionality analyses to identify adverse drug reactions (ADEs) from spontaneous reporting data (Seabroke et al., 2016). Disproportionality analysis mainly serves as a mechanism to formulate hypotheses regarding potential causal connections between pharmaceuticals and their adverse outcomes. This should precede a detailed clinical evaluation of the specific individual case reports involved. The method relies on comparing the observed to the expected frequencies of reports for each specific drug-adverse event pairing (Caster et al., 2020). Therefore, this study used the reporting odds ratio (ROR), the proportional reporting ratio (PRR) (CHEN et al., 2021), the Medicines and Healthcare Products Regulatory Agency (MHRA) (Yanxin et al., 2022), (which also belongs to the PRR, and the difference from the previous PRR is that the thresholds are set differently), the Bayesian confidence propagation neural network (BCPNN) (Zhou et al., 2023) and the multi-item Gamma Poisson Shrinker (MGPS) to tap into the ADE risk signals associated with eculizumab, and the higher the values of the four parameters are, the stronger the signal value (Cui et al., 2023) (Tables 2, 3). In this study signal strength was judged according to 0 < IC-2SD ≤ 1.5 weak signal (+); 1.5 < IC-2SD ≤ 3.0 medium intensity signal (++); 3.0 < IC-2SD high intensity signal (++++) in BCPNN (Guan et al., 2022). The drug label for eculizumab was obtained from Daily Med (https://daily.med.nlm.nih.gov/Daily Med/index.cfm) (Cui et al., 2023).

**TABLE 2:** Two-by-two contingency table for disproportionality analyses.

	Adverse events of interest	All other adverse events of interest	Total
Drug of interest	a	b	a + b
All other drugs of interest	c	d	c + d
Total	a + c	b + d	n = a+ b + c + d

a, Number of reports that contain both targeted drug and targeted drug adverse reactions; b, Number of reports of other drug adverse reactions that contain the targeted drug; c, Number of reports of targeted drug adverse reactions that contain other drugs; d, Number of reports that contain other drugs and other drug adverse reactions.

**TABLE 3:** Four major algorithms used to assess potential associations between eculizumab and ADEs.

Algorithms	Equation	Criteria
ROR	ROR = (ad/bc)	a≥3
	95% CI = eln (ROR)±1.96 (1/a+1/b+1/c+1/d)^0.5	lower limit of 95% CI > 1
PRR	PRR = a (c + d)/c/(a+b)	a ≥3 PRR≥2, χ2≥4
	χ2 = [(ad-bc) ^2] (a+b + c + d)/[(a+b) (c + d) (a+c) (b + d)]	
BCPNN	IC = log2a (a+b + c + d)/((a+c) (a+b))	IC-2SD > 0
	95% CI = E (IC) ± 2 V(IC)^0.5	
MGPS	EBGM = a (a+b + c + d)/(a+c)/(a+b)	EBGM05 > 2
	95% CI = eln (EBGM) ± 1.96 (1/a+1/b+1/c+1/d) ^0.5	

IC-2SD, lower limit of 95% CI of the IC; EBGM05, lower limit of 95% CI of the EBGM.

## 3 Results

### 3.1 Annual distribution of eculizumab-related ADE reports

**FIGURE 2:**
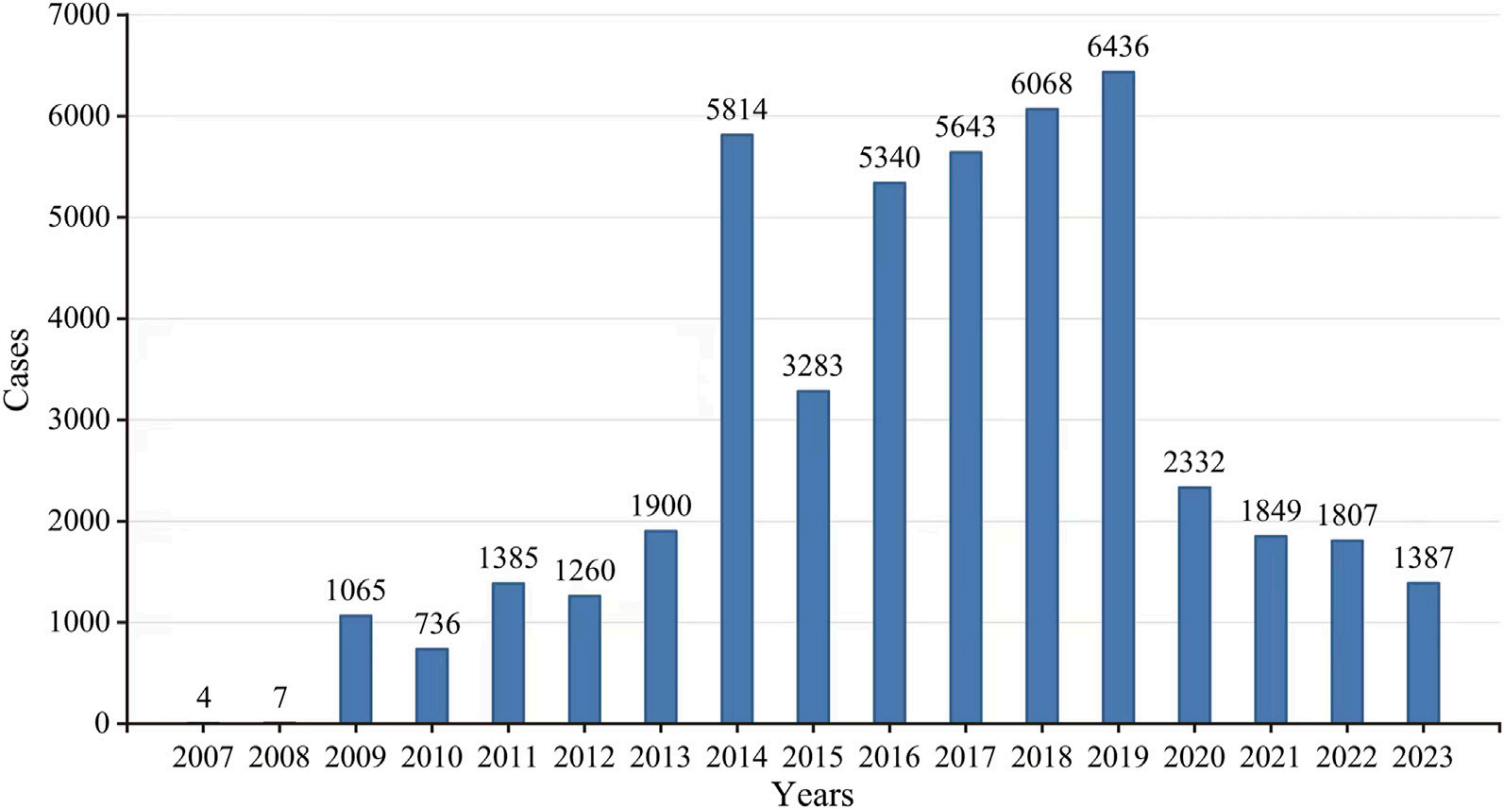
The annual distribution of eculizumab-related ADEs reported from 2007 to 2023.

According to the FAERS database, there were a total of 19,418,776 ADE reports from the first quarter of 2007 to the third quarter of 2023, from which a total of 46,316 ADE reports were screened for eculizumab monotherapy as the first suspected drug. Overall, the number of ADE reports increased substantially in 2014, declined steeply in 2015, showed a small increase from 2016 to 2019, and then declined substantially again in 2020; the number of reports stabilized in the latter years, as the data for 2023 were only counted for three-quarters, and the number of predicted reports did not differ much from that of the previous year; therefore, the number of reports stabilized in the latter years (Figure 2).

### 3.2 General characteristics of the real-world population

Table 4 shows the population characteristics of reports of ADEs associated with eculizumab; of the 46,316 ADE reports, there were more female patients (46.90%) than male patients (31.84%), the age concentration was 18–65 years (17.64%), the source of the reports was predominantly consumers (73.81%), followed by physicians (13.01%). The country of reporting country was the United States (78.91%), and 26,540 (57.30%) serious ADEs were reported, with hospitalization being the main reason (28.25%).

**TABLE 4:** Basic information on the ADE reports related to eculizumab.

Characteristics	Case number (Case proportion, %)
Gender	
Female (%)	21720 (46.90)
Male (%)	14745 (31.84)
Not Specified (%)	9851 (21.27)
Age (years)	
<18 (%)	1733 (3.74)
≥18, <65 (%)	8168 (17.64)
65≤ (%)	2695 (5.82)
Not Specified (%)	33720 (72.80)
Reported Person	
Consumer (%)	34188 (73.81)
Lawyer (%)	2 (0.00)
Not Specified (%)	1736 (3.75)
Other health-professional (%)	3002 (6.48)
Pharmacist (%)	1364 (2.94)
Physician (%)	6024 (13.01)
Reported Countries (top ive)	
United States of America (%)	36548 (78.91)
Brazil (%)	1454 (3.14)
United Kiongdom (%)	1369 (2.96)
Japan (%)	890 (1.92)
Canada (%)	848 (1.83)
Serious reports	
Serious (%)	26540 (57.30)
Non-Serious (%)	19776 (42.70)
Outcome	
Life-Threatening (%)	759 (1.64)
Hospitalization - Initial or Prolonged (%)	13082 (28.25)
Disability (%)	246 (0.53)
Death (%)	4587 (9.90)
Congenital Anomaly (%)	18 (0.04)
Required Intervention to Prevent Permanent	4 (0.01)
Impairment/Damage (%)	
Other (%)	20901 (45.13)
Time to event onset (days)	
0–30d (%)	3069 (6.63)
31–60d (%)	543 (1.17)
61–90d (%)	386 (0.83)
91–120d (%)	246 (0.53)
121–150d (%)	235 (0.51)
151–180d (%)	192 (0.41)
181–360d (%)	908 (1.96)
360d< (%)	3491 (7.54)
Missing or outliers (<0) (%)	37246 (80.42)

### 3.3 Time-to-onset analysis of eculizumab-related ADEs

**FIGURE 3:**
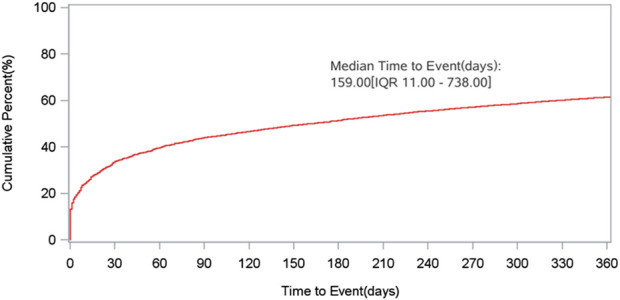
Time to onset of eculizumab-related ADEs.

Onset times for eculizumab-associated ADEs were extracted from the FAERS database and analyzed. After removing all missing or incorrect onset reports, a total of 9070 ADEs with available onset times were included in the analysis. The median onset time was 159 days, with an interquartile range (IQR) of 11–738 days (Figure 3). The time to onset (TTO) of ADEs induced by eculizumab is defined as the interval between EVENT_DT (the date of onset of ADEs in the DEM O file) and S TART_DT (the date of eculizumab initiation in the THER file).

### 3.4 Signal detection at the system organ class level

**FIGURE 4:**
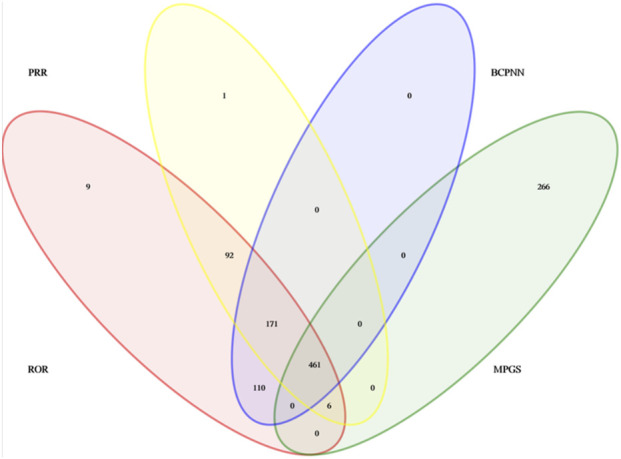
The four methods detect signal crossover when ROR, PRR, BCPNN and MPGS are combined.

**FIGURE 5:**
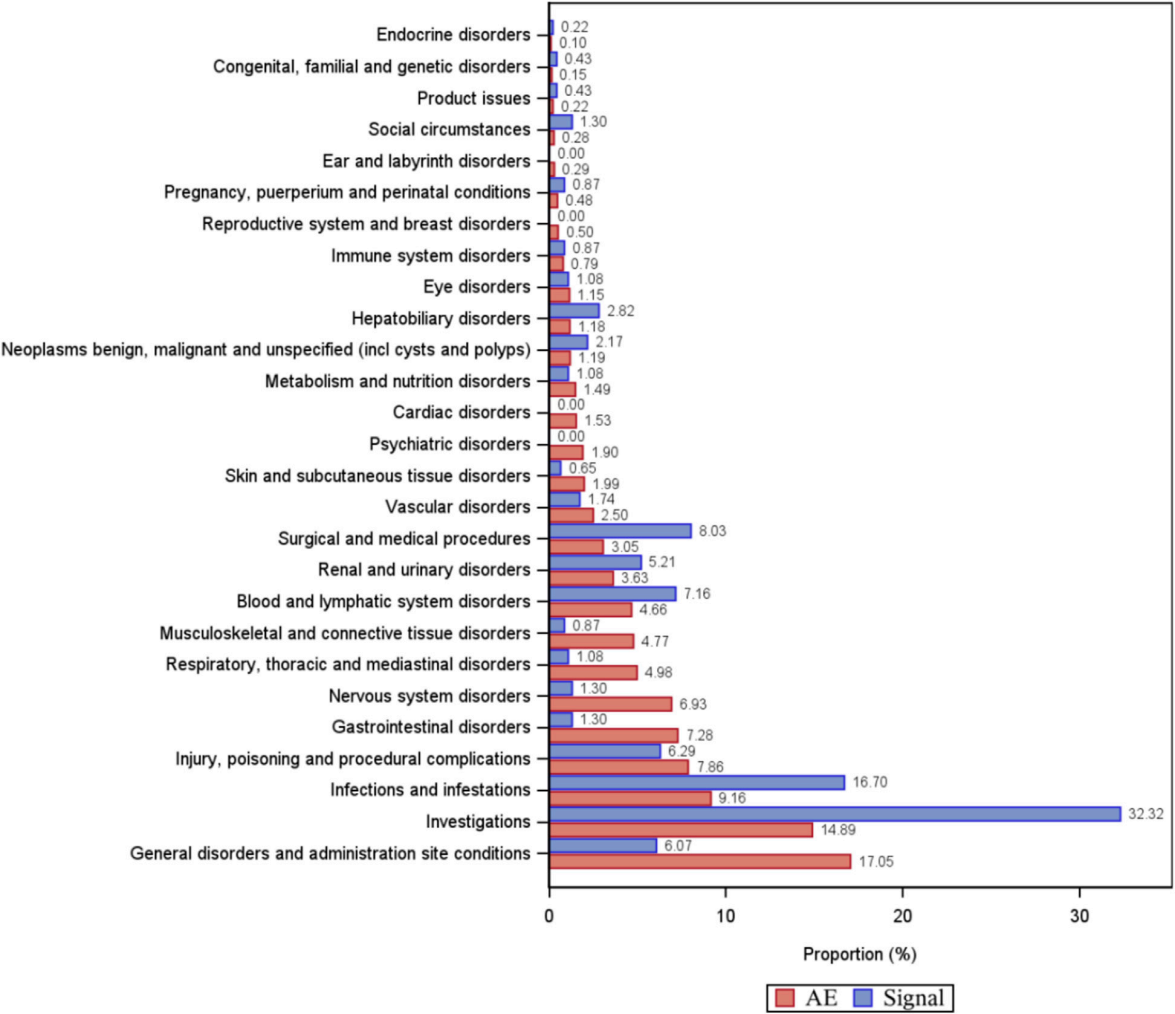
Percentage of the number of PT types and percentage of adverse event cases under organ system classification (SOC). AE, percentage of adverse event cases; Signal, percentage of PT types.

A total of 461 eculizumab-induced ADEs were detected, covering 23 SOCs, in compliance with the four algorithms (Figure 4). The SOCs with the highest percentage of signals were investigated. The SOCs with the greatest number of PTs were general disorders and administration site conditions (Figure 5), with 24,916 patients and 17.05%, respectively. Supplementary Table S1 shows the specific distribution.

### 3.5 Signal detection at the preferred terms level

From 46,316 eculizumab-related ADE reports, 5,436 risk signals (PTs) were mined, and 461 PTs satisfied all four algorithms simultaneously. The complete results are presented in Supplementary Table S2. We ranked the signal strength of all PTs with more than 30 ADE cases (a> 30) based on the value of the EBGM 05 (the most conservative algorithm) (Sakaeda et al., 2013). The distribution of the top 100 signal strengths is shown in Table 5. These PTs reported adverse reactions consistent with the specifications, such as fatigue, nasopharyngitis, back pain, meningococcal infection, fever, and anemia. Some PTs, such as those associated with hemoglobinuria, hemolysis, decreased platelet count, increased blood lactate dehydrogenase, Budd-Chiari syndrome, thrombotic microangiopathy (Sahin et al., 2016; Raina et al., 2019), myasthenia gravis, meuromyelitis optica spectrum disorder, and eyelid ptosis, are closely related to the primary disease. However, attention needs to be paid to whether it is an exacerbation of a preexisting condition, especially in the case of hemolysis and thrombotic microangiopathy, which occur with high frequency and signal strength. According to the instructions, these PTs can still occur after stopping the medication, so attention must still be given to them. Several out-of-specification and clinically significant PTs were also identified, such as aplastic anemia, gene mutation, mastication disorder, kidney fibrosis, BK virus infection, abnormal neutrophil count, C3 glomerulopathy, neuroblastoma, and glomerulonephritis membranoproliferative. In addition, there were some cases of PT with moderate intensity of IC signals, although there were fewer cases, such as EB viraemia [n = 16, IC025 2.81 (1.66)], varicella [n = 27, IC025 2.24 (1.52)], *Neisseria* infections [n = 15, IC025 5.92 (2.89)], gonococcal infections [n = 15, IC025 6.84 (2.98)], *Pseudomonas aeruginosa* sepsis [n = 25, IC025 2.68 (1.83)], and positive tests for norovirus [n = 9, IC025 3.51 (1.55)], which suggests a need for clinical attention. In summary, real data analysis based on the FAERS database can provide a clinical dosing reference for eculizumab.

**TABLE 5:** The top 100 adverse events with respect to signal strength in target drug signal detection.

SOC name	Preferred terms (PTs)	Case numbers	ROR (95% Cl)	PRR	χ2	IC (IC025)	EBGM (EBGM05)
Renal and urinary disorders	Hemoglobinuria	721	799.13 (697.94–915)	795.2	166454.7	7.86 (7.32)	232.15 (202.75)
Blood and lymphatic system disorders	Extravascular hemolysis	175	907.92 (680.71–1210.97)	906.83	41915.36	7.91 (6.38)	240.78 (180.52)
Investigations	Total complement activity decreased*	35	1142.88 (565.95–2307.97)	1142.61	8871.43	7.99 (4.34)	254.69 (126.12)
Infections and infestations	Meningococcal sepsis	71	386.5 (274.07–545.05)	386.31	12497.57	7.47 (5.26)	177.48 (125.85)
Investigations	Haptoglobin abnormal	44	478.95 (301.12–761.81)	478.81	8505.28	7.61 (4.65)	194.71 (122.41)
Renal and urinary disorders	Paroxysmal nocturnal hemoglobinuria	136	269.33 (214.62–337.99)	269.08	19911.79	7.21 (5.86)	147.96 (117.9)
Infections and infestations	Meningococcal bacteraemia	40	450.41 (279.25–726.49)	450.29	7536.52	7.57 (4.51)	189.83 (117.69)
Investigations	Total complement activity increased*	35	1904.81 (801.18–4528.71)	1904.35	9743.76	8.13 (4.34)	279.54 (117.58)
Investigations	Complement factor abnormal*	43	413 (263.38–647.6)	412.88	7801.37	7.51 (4.61)	182.87 (116.62)
Investigations	Haptoglobin decreased*	210	193.39 (163.04–229.39)	193.12	25218.32	6.93 (6.04)	121.71 (102.61)
Investigations	Reticulocyte count increased	209	180.28 (152.26–213.47)	180.03	23983.5	6.86 (6)	116.39 (98.3)
Blood and lymphatic system disorders	Breakthrough hemolysis	41	239.08 (159.8–357.7)	239.02	5610.29	7.11 (4.48)	138.41 (92.51)
Infections and infestations	Meningococcal infection	70	197.1 (146.5–265.17)	197	8513.24	6.95 (5.1)	123.24 (91.6)
Blood and lymphatic system disorders	Hemolysis	1767	137.19 (129.75–145.05)	135.54	166768.11	6.59 (6.43)	96.07 (90.86)
Investigations	Biopsy bone marrow*	128	169.33 (136.77–209.63)	169.18	14095.25	6.8 (5.61)	111.77 (90.28)
Investigations	Blood lactate dehydrogenase abnormal	165	161.94 (134.37–195.18)	161.76	17627.35	6.76 (5.78)	108.5 (90.02)
Surgical and medical procedures	Plasmapheresis*	127	158.38 (128.13–195.78)	158.25	13365.5	6.74 (5.57)	106.91 (86.49)
Infections and infestations	Meningitis meningococcal	37	183.06 (122.4–273.8)	183.02	4291.79	6.88 (4.3)	117.63 (78.65)
Investigations	Reticulocyte count abnormal	30	168.89 (108.69–262.45)	168.86	3299.38	6.8 (4.01)	111.63 (71.84)
Social circumstances	Blood product transfusion dependent*	100	117.09 (93.17–147.15)	117.01	8467.19	6.43 (5.22)	86.4 (68.75)
Investigations	Blood lactate dehydrogenase decreased*	59	107.65 (80.2–144.48)	107.6	4686.51	6.34 (4.7)	81.18 (60.48)
Hepatobiliary disorders	Budd-Chiari syndrome*	45	101.35 (72.53–141.6)	101.32	3411.05	6.28 (4.39)	77.56 (55.51)
Nervous system disorders	Myasthenia gravis crisis*	130	72.36 (59.84–87.51)	72.3	7483.53	5.89 (5.08)	59.37 (49.1)
Metabolism and nutrition disorders	Iron overload*	158	69.22 (58.3–82.18)	69.14	8756.49	5.84 (5.15)	57.23 (48.2)
Investigations	Blood lactate dehydrogenase increased	1548	51.36 (48.67–54.2)	50.83	65442.33	5.46 (5.34)	44.11 (41.8)
Blood and lymphatic system disorders	Intravascular hemolysis	71	61.84 (47.98–79.7)	61.81	3571.48	5.7 (4.56)	52.13 (40.45)
Blood and lymphatic system disorders	Atypical hemolytic uremicc syndrome	84	58.01 (45.99–73.17)	57.98	3994.17	5.63 (4.64)	49.38 (39.15)
Blood and lymphatic system disorders	Aplastic anemia*	495	47.39 (43.13–52.09)	47.24	19572.58	5.37 (5.12)	41.39 (37.66)
Investigations	Red blood cell schistocytes present	35	61.45 (42.82–88.17)	61.43	1751.14	5.7 (3.9)	51.86 (36.14)
Surgical and medical procedures	Red blood cell transfusion	171	43.19 (36.82–50.66)	43.14	6217.67	5.26 (4.74)	38.22 (32.59)
Investigations	Hematocrit abnormal	111	41.78 (34.29–50.91)	41.75	3914.2	5.21 (4.52)	37.13 (30.47)
Investigations	Hemoglobin abnormal	488	36.92 (33.62–40.55)	36.8	15275.95	5.05 (4.82)	33.17 (30.21)
Blood and lymphatic system disorders	Hemolytic uremicc syndrome	180	35.06 (30.06–40.89)	35.02	5372.57	4.99 (4.54)	31.72 (27.2)
Surgical and medical procedures	Platelet transfusion	99	37.09 (30.13–45.66)	37.06	3119.85	5.06 (4.35)	33.39 (27.12)
Surgical and medical procedures	Bone marrow transplant*	133	35.33 (29.54–42.26)	35.3	4000.27	5 (4.44)	31.95 (26.72)
Investigations	Blood creatinine abnormal	222	30.95 (26.97–35.52)	30.91	5869.01	4.82 (4.45)	28.32 (24.68)
Surgical and medical procedures	Transfusion	731	28.96 (26.85–31.24)	28.82	18042.71	4.73 (4.57)	26.56 (24.63)
Investigations	Blood iron increased*	131	31.57 (26.38–37.77)	31.54	3532.55	4.85 (4.31)	28.85 (24.11)
Blood and lymphatic system disorders	Thrombotic microangiopathy	544	27.96 (25.61–30.52)	27.86	12980.6	4.69 (4.49)	25.75 (23.58)
Nervous system disorders	Myasthenia gravis*	329	27.8 (24.84–31.11)	27.74	7816.57	4.68 (4.41)	25.64 (22.91)
Investigations	Blood urea abnormal	52	28.26 (21.28–37.51)	28.25	1257.83	4.7 (3.73)	26.08 (19.64)
Investigations	Platelet count abnormal	268	20.13 (17.8–22.78)	20.1	4582.25	4.25 (3.97)	18.99 (16.79)
Investigations	Mean cell volume increased*	112	21.52 (17.78–26.06)	21.51	2054.81	4.34 (3.83)	20.24 (16.72)
Investigations	Mean cell hemoglobin increased*	72	21.67 (17.07–27.51)	21.66	1330.83	4.35 (3.66)	20.38 (16.05)
Surgical and medical procedures	Renal transplant	151	19.89 (16.87–23.44)	19.87	2550.75	4.23 (3.83)	18.79 (15.94)
Vascular disorders	Poor venous access*	408	18.56 (16.79–20.51)	18.51	6395.46	4.13 (3.93)	17.57 (15.9)
Injury, poisoning and procedural complications	Transfusion reaction*	42	23.17 (16.94–31.68)	23.16	831.59	4.44 (3.42)	21.69 (15.86)
Renal and urinary disorders	Chromaturia	902	16.92 (15.82–18.09)	16.82	12770.11	4 (3.88)	16.05 (15)
Investigations	Hemoglobin decreased	3744	16.42 (15.88–16.97)	16.02	50351.35	3.94 (3.88)	15.32 (14.82)
Surgical and medical procedures	Central venous catheterization	131	18.32 (15.37–21.85)	18.31	2029.72	4.12 (3.69)	17.39 (14.58)
Infections and infestations	Suspected COVID-19*	148	17.42 (14.77–20.56)	17.4	2172.74	4.05 (3.66)	16.57 (14.05)
Injury, poisoning and procedural complications	Renal transplant failure*	43	18.5 (13.61–25.16)	18.5	673.47	4.13 (3.23)	17.56 (12.91)
Investigations	Red blood cell count abnormal	57	17.66 (13.53–23.06)	17.65	849.63	4.07 (3.33)	16.8 (12.87)
Vascular disorders	Malignant hypertension	37	18.82 (13.51–26.21)	18.81	590.11	4.16 (3.15)	17.84 (12.81)
Injury, poisoning and procedural complications	Exposure via body fluid*	48	17.65 (13.2–23.6)	17.65	715.11	4.07 (3.24)	16.79 (12.56)
Investigations	Mean platelet volume decreased	31	17.7 (12.33–25.4)	17.69	463.13	4.07 (2.97)	16.83 (11.73)
Investigations	Hematocrit decreased	568	13.22 (12.16–14.38)	13.17	6144.17	3.67 (3.51)	12.7 (11.68)
Investigations	Serum ferritin increased*	167	14.13 (12.1–16.5)	14.12	1951.13	3.76 (3.43)	13.57 (11.62)
Investigations	Quality of life decreased*	434	13.29 (12.07–14.63)	13.26	4727.18	3.68 (3.5)	12.78 (11.61)
Nervous system disorders	Neuromyelitis optica spectrum disorder*	47	15.97 (11.92–21.41)	15.97	628.63	3.93 (3.13)	15.27 (11.39)
General disorders and administration site conditions	Multimorbidity*	45	14.59 (10.82–19.67)	14.59	545.22	3.81 (3.01)	14.01 (10.39)
Investigations	Blood bilirubin abnormal	40	14.42 (10.5–19.79)	14.41	478.2	3.79 (2.94)	13.85 (10.09)
Congenital, familial and genetic disorders	Gene mutation*	57	12.99 (9.97–16.93)	12.99	606.46	3.65 (3)	12.53 (9.61)
Investigations	Red cell distribution width increased	106	11.19 (9.22–13.58)	11.18	950.53	3.44 (3.03)	10.85 (8.94)
Surgical and medical procedures	Catheter placement	51	10.99 (8.32–14.53)	10.99	448.05	3.41 (2.76)	10.66 (8.07)
Blood and lymphatic system disorders	Bone marrow disorder	69	10.31 (8.11–13.1)	10.3	562	3.32 (2.8)	10.02 (7.88)
Neoplasms benign, malignant and unspecified (incl cysts and polyps)	Myelodysplastic syndrome	290	8.46 (7.53–9.51)	8.45	1856.17	3.05 (2.84)	8.26 (7.35)
Immune system disorders	Transplant rejection*	153	8.37 (7.13–9.83)	8.37	967.7	3.03 (2.73)	8.18 (6.97)
Gastrointestinal disorders	Esophageal spasm*	41	9.75 (7.15–13.3)	9.75	312.58	3.25 (2.53)	9.49 (6.96)
Investigations	Mean cell hemoglobin concentration decreased	36	9.93 (7.13–13.83)	9.93	280.45	3.27 (2.49)	9.66 (6.94)
Surgical and medical procedures	Dialysis	242	7.85 (6.91–8.92)	7.84	1409.69	2.94 (2.71)	7.68 (6.76)
Infections and infestations	Device related infection	311	7.55 (6.75–8.45)	7.54	1724.76	2.89 (2.69)	7.39 (6.61)
Investigations	Platelet count decreased	1739	7.13 (6.79–7.48)	7.05	8860.01	2.79 (2.72)	6.93 (6.6)
Hepatobiliary disorders	Ocular icterus	84	8.27 (6.66–10.27)	8.26	523	3.01 (2.58)	8.08 (6.51)
Surgical and medical procedures	Transplant*	46	8.56 (6.39–11.47)	8.56	299.16	3.06 (2.43)	8.36 (6.24)
Musculoskeletal and connective tissue disorders	Mastication disorder*	56	7.97 (6.11–10.39)	7.97	333.01	2.96 (2.41)	7.8 (5.98)
Investigations	Blood creatinine increased	968	6.47 (6.07–6.9)	6.43	4360.09	2.66 (2.56)	6.33 (5.94)
Surgical and medical procedures	Hemodialysis	126	7.14 (5.99–8.52)	7.14	650.7	2.81 (2.48)	7.01 (5.87)
Immune system disorders	Graft versus host disease*	114	7 (5.82–8.43)	7	573.85	2.78 (2.44)	6.87 (5.71)
Infections and infestations	Viral upper respiratory tract infection	89	7.07 (5.73–8.72)	7.07	453.68	2.79 (2.39)	6.94 (5.62)
Eye disorders	Eyelid ptosis*	152	6.6 (5.62–7.75)	6.6	707.59	2.7 (2.41)	6.49 (5.52)
Hepatobiliary disorders	Jaundice	385	6.14 (5.55–6.79)	6.13	1621.87	2.59 (2.43)	6.03 (5.45)
Blood and lymphatic system disorders	White blood cell disorder*	48	7.39 (5.55–9.84)	7.39	259.43	2.86 (2.27)	7.25 (5.45)
Immune system disorders	Kidney transplant rejection*	75	6.97 (5.55–8.76)	6.97	375.36	2.77 (2.33)	6.84 (5.44)
Renal and urinary disorders	Kidney fibrosis*	32	7.68 (5.41–10.91)	7.68	181.7	2.91 (2.14)	7.53 (5.3)
Investigations	Laboratory test abnormal	410	5.9 (5.35–6.51)	5.89	1635.07	2.54 (2.38)	5.8 (5.26)
Investigations	Blood creatinine decreased*	49	7.03 (5.3–9.33)	7.03	248.18	2.79 (2.21)	6.9 (5.2)
Investigations	Red blood cell count decreased	376	5.64 (5.09–6.25)	5.63	1407.47	2.47 (2.31)	5.55 (5.01)
Infections and infestations	Meningitis	93	6.13 (4.99–7.53)	6.13	391.82	2.59 (2.22)	6.03 (4.91)
Infections and infestations	BK virus infection*	45	6.66 (4.96–8.94)	6.66	211.99	2.71 (2.12)	6.54 (4.87)
Injury, poisoning and procedural complications	Transplant failure*	39	6.79 (4.94–9.32)	6.79	188.52	2.74 (2.08)	6.67 (4.86)
Infections and infestations	Viral infection	397	5.41 (4.9–5.97)	5.4	1399.77	2.41 (2.25)	5.33 (4.82)
Investigations	Blood urea increased	201	5.39 (4.68–6.19)	5.38	705.28	2.41 (2.17)	5.31 (4.62)
Blood and lymphatic system disorders	Platelet disorder	38	6.37 (4.62–8.78)	6.37	168.68	2.65 (2)	6.27 (4.54)
General disorders and administration site conditions	Symptom recurrence*	58	5.96 (4.6–7.73)	5.96	235.23	2.55 (2.06)	5.87 (4.53)
Investigations	Blood urine present	242	5.14 (4.52–5.83)	5.13	792.46	2.34 (2.13)	5.07 (4.46)
Hepatobiliary disorders	Portal vein thrombosis	44	5.92 (4.4–7.98)	5.92	176.76	2.54 (1.96)	5.83 (4.33)
Infections and infestations	Bacteraemia	135	5.18 (4.37–6.14)	5.18	448.03	2.35 (2.06)	5.11 (4.31)
General disorders and administration site conditions	Catheter site pain*	34	6.01 (4.28–8.43)	6.01	139.32	2.56 (1.88)	5.92 (4.21)
Investigations	Neutrophil count abnormal*	37	5.92 (4.28–8.19)	5.92	148.54	2.54 (1.9)	5.83 (4.21)

Note: PTs with * indicate that they are not included in the specification.

### 3.6 PT distribution of key SOCs

Since most of the risk signals of the SOCs in the first and second orders of reporting were related to the primary disease treated with eculizumab, the SOCs in the third and fourth orders of reporting (injury, poisoning and procedural complications, infections and infestations), as well as immune disorders of clinical concern and benign, malignant and tumors of undetermined nature (including cystic and polypoid), were selected as the key SOCs to be analyzed in the present study. Table 6 shows the distribution of the top 10 PTs in terms of frequency of occurrence under each of the above SOCs, and PTs in immune system diseases and benign, malignant, and tumors of unknown nature were not included in the manual.

**TABLE 6:** PT distribution of eculizumab-related ADE reports focused on SOCs.

SOC	PT	N	ROR (95% CI)	PRR (χ2)	IC(IC025)	EBGM(EBGM05)
Injury, poisoning and procedural complications	Inappropriate schedule of product administration^a^	1569	2.86 (2.72–3)	2.84 (1857.05)	1.5 (1.42)	2.82 (2.68)
	Incorrect dose administered^a^	1112	2.36 (2.23–2.51)	2.35 (861.5)	1.23 (1.14)	2.34 (2.21)
	Exposure during pregnancy^a^	554	2.59 (2.38–2.82)	2.58 (534.53)	1.36 (1.24)	2.57 (2.37)
	Maternal exposure during breast feeding^a^	60	3.55 (2.76–4.58)	3.55 (108.89)	1.82 (1.39)	3.53 (2.73)
	Exposure via body fluid^a^	48	17.65 (13.2–23.6)	17.65 (715.11)	4.07 (3.24)	16.79 (12.56)
	Renal transplant failure^a^	43	18.5 (13.61–25.16)	18.5 (673.47)	4.13 (3.23)	17.56 (12.91)
	Transfusion reaction^a^	42	23.17 (16.94–31.68)	23.16 (831.59)	4.44 (3.42)	21.69 (15.86)
	Transplant failure^a^	39	6.79 (4.94–9.32)	6.79 (188.52)	2.74 (2.08)	6.67 (4.86)
	Arteriovenous fistula site complication	28	16.81 (11.49–24.57)	16.8 (395.79)	4 (2.85)	16.03 (10.96)
	Complications of transplanted kidney^a^	27	5.51 (3.77–8.06)	5.51 (97.99)	2.44 (1.68)	5.43 (3.71)
Infections and infestations	Nasopharyngitis	1240	2.89 (2.73–3.05)	2.87 (1503.91)	1.51 (1.43)	2.86 (2.7)
	Infection	738	2.24 (2.09–2.41)	2.24 (503.09)	1.16 (1.05)	2.23 (2.07)
	Sepsis	640	2.41 (2.23–2.61)	2.41 (523.53)	1.26 (1.14)	2.4 (2.22)
	Influenza	586	2.34 (2.16–2.54)	2.34 (444.97)	1.22 (1.09)	2.33 (2.14)
	Viral infection	397	5.41 (4.9–5.97)	5.4 (1399.77)	2.41 (2.25)	5.33 (4.82)
	Upper respiratory tract infection	320	2.96(2.65–3.31)	2.96 (411.16)	1.56 (1.39)	2.94 (2.63)
	Device related infection^a^	311	7.55 (6.75–8.45)	7.54 (1724.76)	2.89 (2.69)	7.39 (6.61)
	Lower respiratory tract infection	231	2.39 (2.1–2.72)	2.39 (185.73)	1.25 (1.05)	2.38 (2.09)
	Gastroenteritis viral	164	3.94 (3.37–4.59)	3.93 (354.45)	1.96 (1.71)	3.9 (3.34)
	Suspected COVID-19^a^	148	17.42 (14.77–20.56)	17.4 (2172.74)	4.05 (3.66)	16.57 (14.05)
Neoplasms benign, malignant and unspecified (incl cysts and polyps)	Myelodysplastic syndrome	290	8.46 (7.53–9.51)	8.45 (1856.17)	3.05 (2.84)	8.26 (7.35)
	Acute leukemia^a^	20	5.1 (3.28–7.93)	5.1 (64.93)	2.33 (1.44)	5.04 (3.24)
	Marrow hyperplasia^a^	15	9.89 (5.92–16.54)	9.89 (116.38)	3.27 (1.91)	9.63 (5.76)
	Neuroblastoma^a^	10	11.87 (6.32–22.31)	11.87 (96.06)	3.52 (1.67)	11.49 (6.11)
	Thymoma^a^	7	9.18 (4.33–19.45)	9.18 (49.61)	3.16 (1.13)	8.95 (4.22)
	Myelodysplastic syndrome with single lineage dysplasia^a^	6	5.95 (2.66–13.35)	5.95 (24.29)	2.55 (0.69)	5.86 (2.62)
	Pancreatic carcinoma recurrent^a^	6	13.24 (5.85–29.94)	13.23 (65.22)	3.67 (1.13)	12.76 (5.64)
	Castleman’s disease^a^	5	5.75 (2.37–13.92)	5.75 (19.27)	2.5 (0.48)	5.67 (2.34)
	Clonal evolution^a^	3	11.39 (3.6–36.01)	11.39 (27.47)	3.46 (0.18)	11.04 (3.49)
	Angiolipoma^a^	3	17.81 (5.57–56.92)	17.81 (45.13)	4.08 (0.27)	16.94 (5.3)
Immune system disorders	Transplant rejection^a^	153	8.37 (7.13–9.83)	8.37 (967.7)	3.03 (2.73)	8.18 (6.97)
	Graft versus host disease^a^	114	7 (5.82–8.43)	7 (573.85)	2.78 (2.44)	6.87 (5.71)
	Kidney transplant rejection^a^	75	6.97 (5.55–8.76)	6.97 (375.36)	2.77 (2.33)	6.84 (5.44)
	Alloimmunization^a^	3	30.61 (9.37–99.95)	30.61 (78.55)	4.81 (0.33)	28.07 (8.59)

Note: If there are fewer than 10 PTs with signals in this table, all PTs, will be exhibited.

^a^ Indicates adverse reactions not included in the specification; “N” indicates the number of repor.

### 3.7 Subgroup analysis

#### 3.7.1 Age subgroup

**FIGURE 6:**
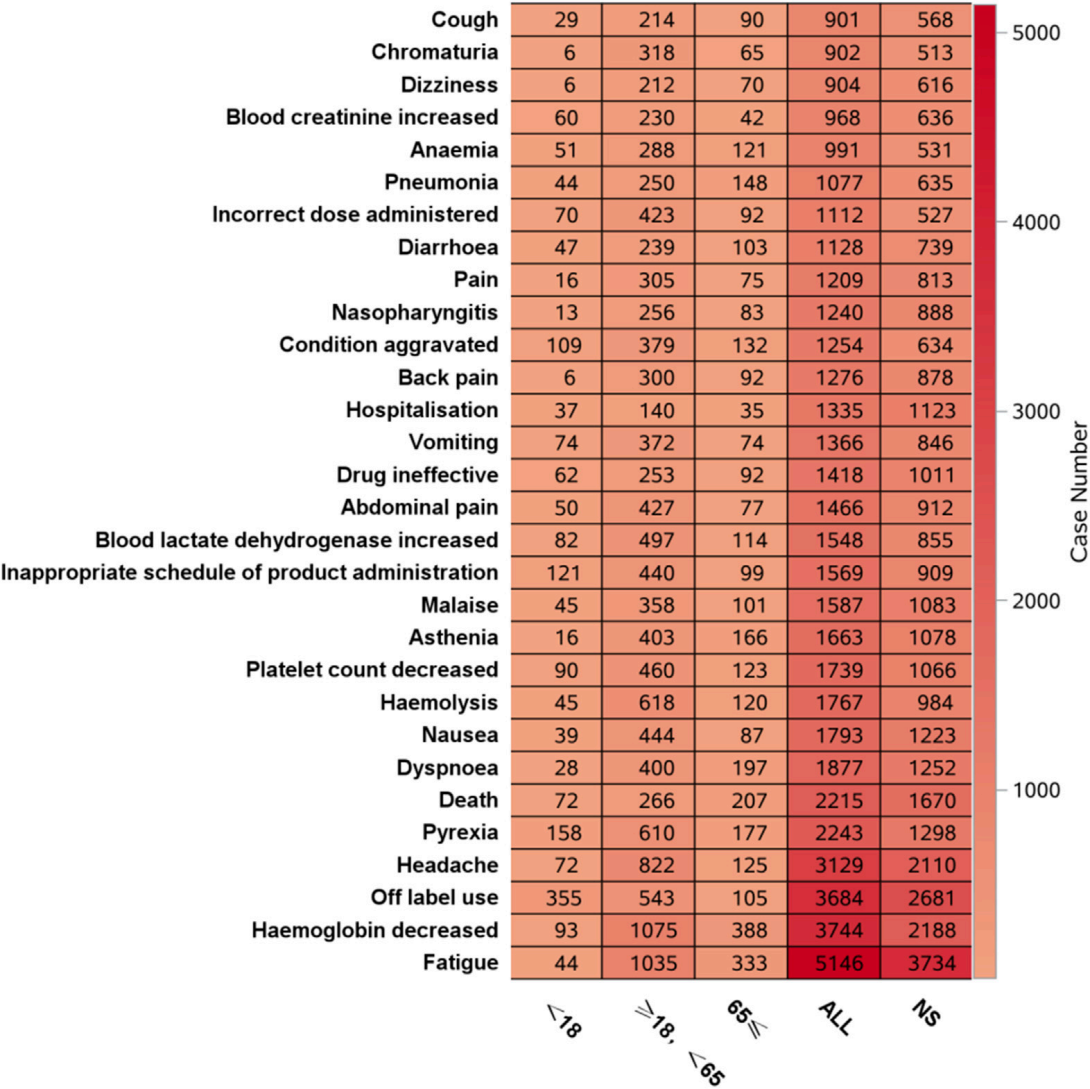
Age-based subgroup analysis of the frequency of eculizumab-related ADEs.

**FIGURE 7:**
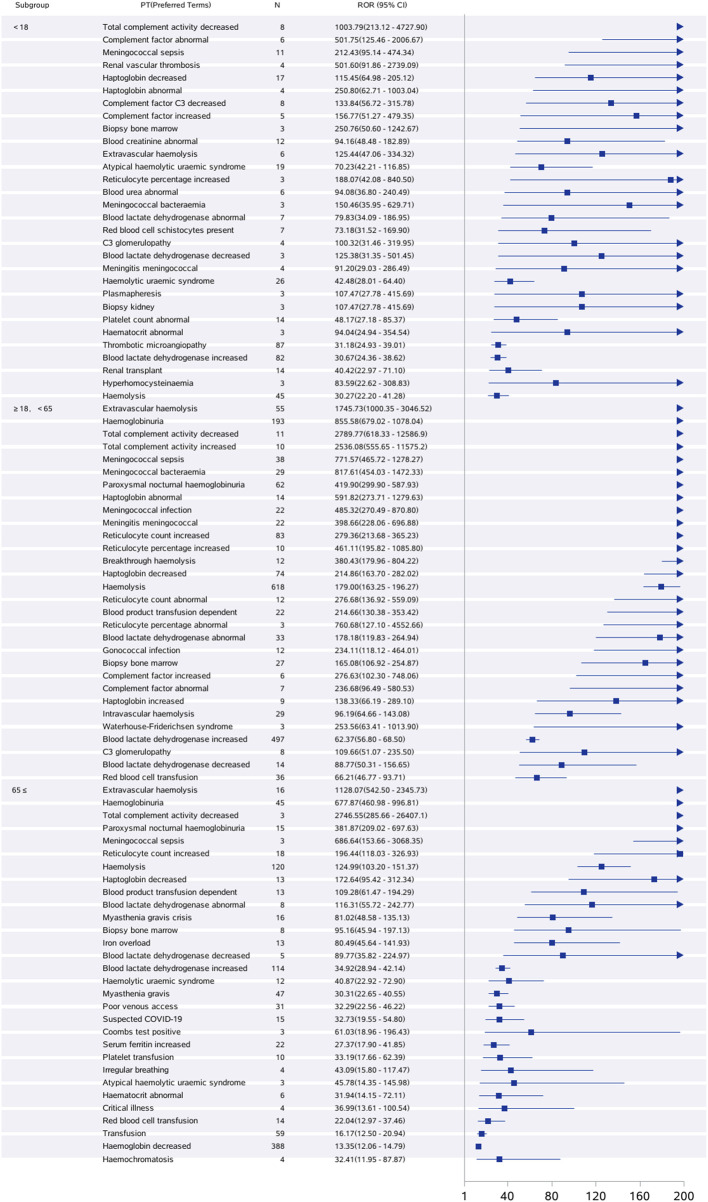
Age-based subgroup analysis of eculizumab-associated ADE signal intensity.

Figure 6 shows that the most frequent PT in the <18 years age group was “off-label use”, and in the ≥18 years, <65 years age group and ≥65 years age group, the most frequent PT was “Hemoglobin decreased”. Figure 7 shows the top 30 orders of signal strength by age, calculated as the ROR (95% CI). In the <18-year-old group, the strong signals were decreased total complement activity, abnormal complement factors, meningococcal sepsis, renal vascular thrombosis, and reduced binding bead protein. In the ≥18-year-old group, the signals were strong for extravascular hemolysis, hemoglobinuria, decreased total complement activity, increased total complement activity, meningococcal sepsis, and meningococcal bacteremia. In the ≥65-year-old group, the signals were strong for extravascular hemolysis, decreased hemoglobinuria, decreased total complement activity, paroxysmal nocturnal hemoglobinuria, meningococcal sepsis, and elevated reticulocyte count. Meningococcal sepsis, which had the highest signal intensity in all three groups, had the highest signal intensity, with a ROR (95% CI) of 771.57 (465.72-1278.27) in the ≥18-year-old group, <65-year-old group, and three cases of the Waterhouse-Friderichsen syndrome, which were not reported in the other two groups. In the <18 years group, hyperhomocysteinemia was found to be overspecified.

#### 3.7.2 Gender differences in eculizumab-associated ADEs

**FIGURE 8:**
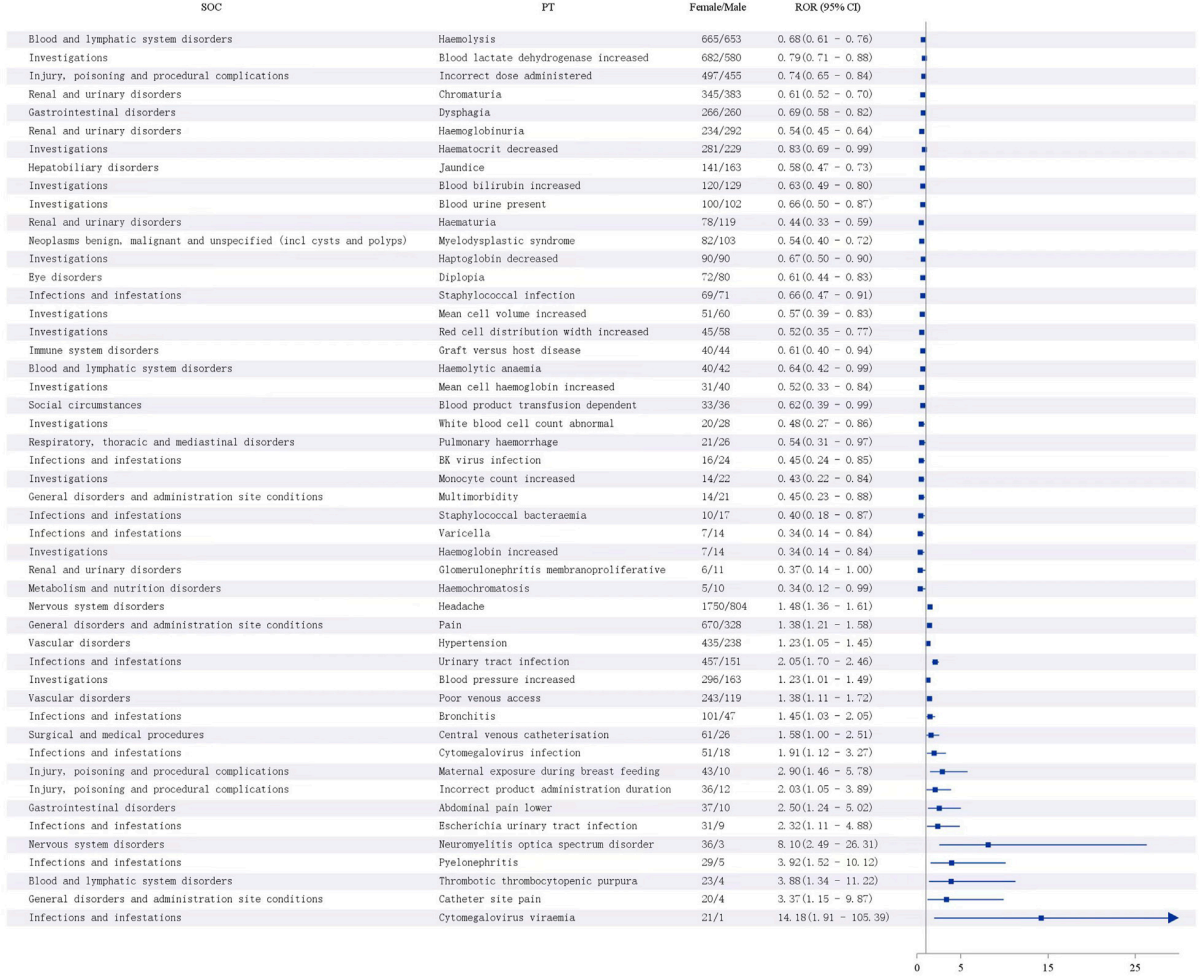
Analysis of sex differences in Eculizumab-associated ADEs.

At the PT level, using the ROR and a ≥3; ROR >1, the lower limit of the 95% CI > 1, suggests that female patients are more likely than male patients to report a particular ADE, and the larger the value is, the stronger the association, and ROR <1, the upper limit of the 95% CI < 1; thus, male patients are more likely to report that the smaller the value of the ADE is, the stronger the association (Bate and Evans, 2009). A total of 51 PTs were identified among the 461 PTs, revealing an imbalance in ADE occurrence between males and females. Headache, pain, hypertension, urinary tract infection, poor venous access, bronchitis, cytomegalovirus infection, and thrombotic thrombocytopenic purpura were more likely to occur in females, and hemolysis, elevated blood lactate dehydrogenase, dysphagia, myelodysplastic syndromes, graft-versus-host disease, abnormal white blood cell counts, pulmonary hemorrhage, BK virus infection, staphylococcal bacteremia, chickenpox, elevated hemoglobin, and membranoproliferative glomerulonephritis were more likely to occur in males (Figure 8).

## 4 Discussion

We conducted a postmarketing pharmacovigilance analysis of eculizumab by collecting and evaluating real-world data from the largest sample to identify potential new adverse reactions to eculizumab and analyze the timing of adverse reactions and sex differences. The results of this study will help guide the safe clinical use of this drug.

### 4.1 Infection-related adverse reactions

The most common symptom under this SOC was nasopharyngitis, and the strongest signals were meningococcal bacteremia and meningococcal line septicemia. One study included 131 patients for analysis, including 107 patients with *Neisseria meningitidis* infection (81.7%), ten patients with *Neisseria* gonorrhea infection (7.7%), and patients with infections caused by other pathogens, including other genera of *Neisseria*, Cryptococcus, *Aspergillus niger*, *Staphylococcus*, *P. aeruginosa*, *Clostridium difficile*, and varicella-zoster virus (Li-li et al., 2022b). Fatal meningococcal infections occurred in patients treated with Soliris. The Advisory Committee on Immunization Practices (ACIP) encourages patients treated with eculizumab to receive vaccines against serotypes A, C, W, Y, and B, which should be given to patients at least 2 weeks before the first dose of Soliris is given. However, between 2008 and 2016, 16 cases of meningococcal disease were identified in patients treated with eculizumab in the United States; 11 of these cases were caused by ungroupable *Neisseria* meningitidis. Fourteen patients were documented to have received at least one dose of meningococcal vaccine before the onset of disease (McNamara et al., 2017). Because meningococcal vaccination may not prevent all cases of meningococcal infection in eculizumab-treated patients, the Centers for Disease Control and Prevention recommends that appropriate antibiotic prophylaxis be considered; all patients should be monitored for early signs of meningococcal infection (Socié et al., 2019).

### 4.2 Injury, poisoning and procedural complications

The most frequent occurrences were products given at the wrong time and incorrect doses. According to the Soliris instruction manual, the recommended dose and duration of administration for the treatment of PNH are 600 mg weekly for the first 4 weeks, followed by a fifth dose of 900 mg and thereafter 900 mg every 2 weeks. In 2013, eculizumab was approved in Japan for the treatment of complement-mediated aHUS at a dosage of 900 mg weekly for 4 weeks in adult patients, followed by 1,200 mg maintenance in week five and then 1,200 mg maintenance every 2 weeks (TianQi and XiaoWen, 2020). The recommended mode of administration in the instructions is intravenous infusion. The optimal duration of eculizumab therapy in patients with atypical hemolytic uremic syndrome (aHUS) is still unclear, but several prospective studies have shown that in patients with aHUS, it is safe to discontinue eculizumab once a complete remission has been achieved in the majority of patients with atypical hemolytic uremic syndrome (aHUS) (Fakhouri et al., 2021; Brodsky, 2021). This requires that we, as healthcare professionals, manage the duration of treatment to minimize the incidence of adverse events and the cost of treatment. Second, 548 cases of exposure during pregnancy have been reported, with studies showing that eculizumab was not present in breast milk and that the levels observed in cord blood samples were insufficient to affect neonatal complement concentrations. Eculizumab may be considered safe in pregnancy, but due to the paucity of safety data, it is still not possible to completely exclude mothers and fetuses from treatment for PNH, aHUS, and HELLP syndrome (Sarno et al., 2019).

### 4.3 Adverse reactions associated with various neurological disorders

Among the various neurological disorders, the most frequent were headache (3119 cases), myasthenia gravis, myasthenia gravis crisis, optic neuromyelitis optica spectrum disorders, cerebral thrombosis and cerebral venous thrombosis, although not included in the eculizumab specification, which were associated with the primary disease. It is also worth noting that patients with refractory generalized myasthenia gravis treated with eculizumab may experience worsening symptoms (Howard et al., 2017). Patients should be informed of the possibility of these risk signals when using the drug clinically.

### 4.4 Tumor-related adverse reactions

The Eculizumab instructions mention malignant melanoma and myelodysplastic syndromes as rare. The top 10 PTs in terms of ADE reports are shown in Table 6, with the majority of PTs not included in the instructions. It has been shown that patients with PNH on eculizumab report more hematological tumors than patients with aHUS, with a reporting rate of approximately 5.0 cases per 12 years, and with regard to solid tumors, skin tumors were more common in patients with PNH (74% solid tumors) than in patients with aHUS (100% solid tumors), with the rate of reporting of solid tumors remaining stable over time; at approximately 5.0 cases per 15 years, hematological tumors were leukemia and lymphoma, and solid tumors were gastrointestinal, skin, genital, breast, and others (respiratory, hepatobiliary, central nervous system, renal; <5% each) (Socié et al., 2019). For safety reasons, patients using eculizumab should be monitored for tumorigenesis, and eculizumab should be used with caution in patients with a history of malignancy or in patients who develop malignancy.

### 4.5 Adverse reactions associated with immune system disorders

As with all proteins, eculizumab is potentially immunogenic, and antibodies to eculizumab were detected in 2% of patients with PHN using ELISA and in 3% of patients with aHUS and 2% of patients with NMOSD using enhanced chemiluminescence (ECL)-bridged immunogenicity analysis. In this real-world study, only four PTs were screened from 5,436 PTs for relevant immune system disorders that met the four algorithms: transplant rejection, graft-versus-host disease, renal transplant rejection, and allogeneic rejection. Graft-versus-host disease (GVHD) is a major complication of allogeneic hematopoietic cell transplantation (HCT) (Toubai et al., 2008). Patients with AA/PNH (plastic anemia/paroxysmal nocturnal hemoglobinuria) syndrome may need to be “transitioned” to treatment with eculizumab before bone marrow transplantation to reduce the risk of intravascular hemolysis and thrombosis (DeZern et al., 2018). A previous report examining the outcomes of 21 patients treated with HSCT (hematopoietic stem cell transplantation) between 2007 and 2017 who had received prior treatment with eculizumab revealed that HSCT still had a mortality rate of nearly 30%, mainly due to infection and acute graft-versus-host disease (GvHD) (Vallet et al., 2018).

### 4.6 Timing of the onset of adverse effects and sex differences

The median time to onset of eculizumab adverse events analyzed in our study was 159 days, with an interquartile range (IQR) of 11∼738 days. Early and timely recognition and management of eculizumab treatment-induced adverse events are critical. A study showed that women are more likely to suffer adverse drug reactions than men (Zopf et al., 2009). This was also confirmed in the present study, where women were more likely than men to have the same positive signal value.

### 4.7 Eculizumab biosimilars

The clinical safety of eculizumab biosimilars is paramount. Two such biosimilars have been identified: Bkemv (eculizumab-aeeb) and Epysqli (eculizumab-aagh). On 29 May 2024, the U.S. FDA granted approval to Amgen’s Bkemv as the first biosimilar interchangeable with AstraZeneca’s Soliris (eculizumab), used for reducing hemolysis in patients with paroxysmal nocturnal hemoglobinuria (PNH) and tinhibiting complement-mediated thrombotic microangiopathy in patients with atypical hemolytic uremic syndrome (aHUS). Epysqli, manufactured by Samsung Bioepis Co. Ltd., obtained initial approval in the European Union, Iceland, Liechtenstein, and Norway on 26 May 2023, and later received FDA approval on 19 July 2024. Adverse reaction data on eculizumab were gathered from the FAERS database from the first quarter of 2007 to the third quarter of 2023, prior to FDA approval of these biosimilars. The analysis timeframe was extended to include post-approval adverse reactions related to the biosimilars; however, due to the recent approvals, no data is currently available. Additionally, the Vigibase database, which was searched without time constraints, revealed 51,275 adverse reaction reports involving eculizumab’s active ingredient. However, the database does not specify whether the reactions were linked to the original drug or a biosimilar, thus no specific reports for biosimilars were identified. Although no safety data for the biosimilars were found, ongoing surveillance will continue. Literature on adverse reactions to generic drugs was also reviewed. In a Phase III, randomised, double-blind, multi-national clinical trial comparing SB12 (the proposed eculizumab biosimilar) with the reference eculizumab in patients with paroxysmal nocturnal haemoglobinuria (PNH), treatment-emergent adverse events were reported in 72% of patients in the SB12 treatment group and 68% in the ECU treatment group, respectively. The results demonstrate equivalence between SB12 and ECU and support the use of SB12 in PNH patients (Jang et al., 2023). With the FDA approval of eculizumab biosimilars, they will be widely used and in-depth studies of their safety and equivalence would be needed.

Despite the advantages of data mining, FAERS, as a passive surveillance system, has many limitations. First, it is not possible to determine whether the reported events are related to drugs; at the same time, the reported information is usually insufficient and difficult to evaluate accurately. In addition, the FAERS cannot be used to calculate the incidence of ADEs in the population because of duplicated reports, underreporting, etc. (DONG et al., 2017). Therefore, the Eculizumab ADE risk signals revealed in this study need to be confirmed by high-quality, multicenter clinical studies. Among other things, prospective studies can then test any hypotheses derived using pharmacovigilance databases.

## 5 Conclusion

In this study, a total of 46,316 reports of adverse drug events (ADE) caused by eculizumab monotherapy as the first suspected drug were screened through signal mining in the FAERS database, and 461 PTs satisfying the four algorithms were identified at the same time, covering 23 SOCs, which were scientifically and systematically analyzed at the level of organ classification and PT, as well as disease onset time and differences in sex and age. This study provides a reference for clinical drug safety.

## Data Availability

The datasets presented in this study can be found in online repositories. The names of the repository/repositories and accession number(s) can be found in the article/Supplementary Material.

## Abbreviations

ADE, Adverse drug events; FAERS, Food and Drug Administration Adverse Event Reporting System; ROR, Reporting Odds Ratio; PRR, Proportional Reporting Ratio; MHRA, Medicines and Healthcare Products Regulatory Agency; ASCII, American Standard Code for Information Interchange; BCPNN, Bayesian Confidence Propagation Neural Network; MGPS, Muti-item Gamma Poisson Shrinker; PT, Preferred Terms; SOC, System Organ Class; IQR, Interquartile range; PNH, paroxysmal nocturnal hemoglobinuria; aHUS, atypical hemolytic uremic syndrome; gMG, generalized myasthenia gravis; AQP4-IgG+NMOSD, aquaporin-4 immunoglobulin G-positive optic neuromyelitis optica spectrum disorders; DEMO, demographic and management information; MedDRA, Medical Dictionary for Regulatory Activities; PS, Primary Suspect; HSCT, hematopoietic stem cell transplantation; FDA, The Food and Drug Administration.
