# Corrigendum: Adverse drug events (ADEs) risk signal mining related to eculizumab based on the FARES database

**DOI:** 10.3389/fphar.2025.1558930

**Published:** 2025-02-07

**Authors:** Xi-Feng Wang, Lu-Ri Bao, Ta-La Hu, Rui-Feng Xu, Wu-Niri Gao, Jing-Yuan Wang, Jian-Rong Zhao, Zhen-Li Fu, Shu-Fang Wang, Yan Meng

**Affiliations:** ^1^ Department of Nephrology, The Affiliated Hospital of Inner Mongolia Medical University, Hohhot, China; ^2^ Department of Pathology, School of Basic Medicine, Inner Mongolia Medical University, Hohhot, China; ^3^ Department of Hemodialysis, The No. 2 Hospital of Hohhot, Hohhot, China; ^4^ The First Department of Specialty Medicine, Inner Mongolia Corps Hospital of The Chinese People’s Armed Police Force, Hohhot, China

**Keywords:** eculizumab, FAERS, adverse drug events, adverse drug reaction monitoring, ADRM

In the published article, there was an error in the **Author list**, and the order of authors “Yan Meng” and “Shu-Fang Wang” was incorrect. The corrected author list appears below.

“Xi-Feng Wang^1^, Lu-Ri Bao^2^, Ta-La Hu^1^, Rui-Feng Xu^1^, Wu-Niri Gao^1^, Jing-Yuan Wang^2^, Jian-Rong Zhao^1^, Zhen-Li Fu^3^, Shu-Fang Wang^4^* and Yan Meng^1^*”.

The authors apologize for this error and state that this does not change the scientific conclusions of the article in any way. The original article has been updated.

