# Supplementary material for: Adverse drug events (ADEs) risk signal mining related to eculizumab based on the FARES database
Source: Front Pharmacol. 2025 Jan 9;15:1440907. doi: 10.3389/fphar.2024.1440907 (PMC11754194; doi:10.3389/fphar.2024.1440907)
Supplement: Supplementary file 2 [file Table1.docx]

|  | **Signal** | | **AE** | |
| --- | --- | --- | --- | --- |
| **System Organ Class（SOC）** | **Signal number** | **Proportion** | **N** | **Proportion** |
| General disorders and administration site conditions | 28 | 6.07 | 24916 | 17.05 |
| Investigations | 149 | 32.32 | 21764 | 14.89 |
| Infections and infestations | 77 | 16.70 | 13379 | 9.16 |
| Injury, poisoning and procedural complications | 29 | 6.29 | 11487 | 7.86 |
| Gastrointestinal disorders | 6 | 1.30 | 10634 | 7.28 |
| Nervous system disorders | 6 | 1.30 | 10121 | 6.93 |
| Respiratory, thoracic and mediastinal disorders | 5 | 1.08 | 7270 | 4.98 |
| Musculoskeletal and connective tissue disorders | 4 | 0.87 | 6974 | 4.77 |
| Blood and lymphatic system disorders | 33 | 7.16 | 6810 | 4.66 |
| Renal and urinary disorders | 24 | 5.21 | 5302 | 3.63 |
| Surgical and medical procedures | 37 | 8.03 | 4463 | 3.05 |
| Vascular disorders | 8 | 1.74 | 3649 | 2.50 |
| Skin and subcutaneous tissue disorders | 3 | 0.65 | 2904 | 1.99 |
| Psychiatric disorders | 0 | 0.00 | 2781 | 1.90 |
| Cardiac disorders | 0 | 0.00 | 2240 | 1.53 |
| Metabolism and nutrition disorders | 5 | 1.08 | 2178 | 1.49 |
| Neoplasms benign, malignant and unspecified (incl cysts and polyps) | 10 | 2.17 | 1736 | 1.19 |
| Hepatobiliary disorders | 13 | 2.82 | 1720 | 1.18 |
| Eye disorders | 5 | 1.08 | 1682 | 1.15 |
| Immune system disorders | 4 | 0.87 | 1161 | 0.79 |
| Reproductive system and breast disorders | 0 | 0.00 | 734 | 0.50 |
| Pregnancy, puerperium and perinatal conditions | 4 | 0.87 | 696 | 0.48 |
| Ear and labyrinth disorders | 0 | 0.00 | 429 | 0.29 |
| Social circumstances | 6 | 1.30 | 408 | 0.28 |
| Product issues | 2 | 0.43 | 321 | 0.22 |
| Congenital, familial and genetic disorders | 2 | 0.43 | 218 | 0.15 |
| Endocrine disorders | 1 | 0.22 | 149 | 0.10 |
| Total | 461 | 100.00 | 146126 | 100.00 |

Table S1 Distribution of target adverse drug events in different system organ
